# Solar-Driven Producing of Value-Added Chemicals with Organic Semiconductor-Bacteria Biohybrid System

**DOI:** 10.34133/2022/9834093

**Published:** 2022-03-23

**Authors:** Wen Yu, Haotian Bai, Yue Zeng, Hao Zhao, Shengpeng Xia, Yiming Huang, Fengting Lv, Shu Wang

**Affiliations:** ^1^Beijing National Laboratory for Molecular Sciences, Key Laboratory of Organic Solids, Institute of Chemistry, Chinese Academy of Sciences, Beijing 100190, China; ^2^College of Chemistry, University of Chinese Academy of Sciences, Beijing 100049, China

## Abstract

Photosynthetic biohybrid systems exhibit promising performance in biosynthesis; however, these systems can only produce a single metabolite and cannot further transform carbon sources into highly valuable chemical production. Herein, a photosynthetic biohybrid system integrating biological and chemical cascade synthesis was developed for solar-driven conversion of glucose to value-added chemicals. A new ternary cooperative biohybrid system, namely bacterial factory, was constructed by self-assembling of enzyme-modified light-harvesting donor-acceptor conjugated polymer nanoparticles (D-A CPNs) and genetically engineered *Escherichia coli* (*E. coli*). The D-A CPNs coating on *E. coli* could effectively generate electrons under light irradiation, which were transferred into *E. coli* to promote the 37% increment of threonine production by increasing the ratio of nicotinamide adenine dinucleotide phosphate (NADPH). Subsequently, the metabolized threonine was catalyzed by threonine deaminase covalently linking with D-A CPNs to obtain 2-oxobutyrate, which is an important precursor of drugs and chemicals. The 2-oxobutyrate yield under light irradiation is increased by 58% in comparison to that in dark. This work provides a new organic semiconductor-microorganism photosynthetic biohybrid system for biological and chemical cascade synthesis of highly valuable chemicals by taking advantage of renewable carbon sources and solar energy.

## 1. Introduction

Microorganisms have been attractive in the fields of microbial synthesis on account of their ability to convert renewable carbon sources into high-value chemicals through extensive metabolism pathways [1, 2]. It is well known that microorganisms can use carbon sources such as CO_2_ or glucose for biosynthesis through metabolic pathways of Calvin cycle and tricarboxylic acid cycle [3–5]. However, low synthetic efficiency and complicated by-products severely restrict the development of microbial synthesis. Scientists have attempted to solve these key issues through biological and chemical methods. For example, genetic engineering can enhance the synthesis precision of microorganisms by utilizing genetic tools to modify intracellular metabolism and create new ways beyond the native metabolic models [6]. Although the priorities have already been achieved by genetic engineering, the sophisticated laboratory operations limited its wide applications. Alternatively, the photosynthetic biohybrid systems combining microorganisms with photoelectric-active materials are another promising approach to improve the microbial synthesis efficiency, where generating electrons from photoelectric-active materials under light irradiation can be injected into microorganisms to promote the ratios of effective intermediates, so that transforming renewable carbon sources into more valuable chemicals [7, 8].

Semiconductor-microorganism photosynthetic biohybrid systems [8–11], due to their ability of synthesizing complex chemicals [12], can provide sustainable biosynthesis for expanding storage of solar energy and convert solar energy into chemical energy by H_2_ production [13–16], photosynthetic enhancement [17], CO_2_ reduction [1, 18–20], and N_2_ fixation [21–23]. However, the current biohybrid photosynthetic systems can only rely on the existing metabolic pathways of microorganisms to produce a single metabolite and cannot further transform carbon sources into complex target substances. Importantly, chemical synthesis is the most artificial synthesis pathway to produce desirable chemicals on demand by catalysts or enzymes, which greatly improves the synthesis precisions. Thus, it is anticipated to fabricate a photosynthetic semiconductor-microorganism biohybrid system integrating biological and chemical cascade synthesis to improve the synthesis precision and efficiency. Conducting polymer nanoparticles (CPNs) possess good biocompatibility, excellent light-harvesting and photo-electronic conversion capabilities [24, 25], and adjustable orbital energy levels, so CPNs have been utilized to enhance the electron transmission for reducing CO_2_ to acetic acid and expand the absorption spectra of algae [26–28]. In addition, CPNs are featured with large surface area and active modification groups, which are easy to interact with biological agents including antibodies and enzymes [29].Thus, the construction of photosynthetic biosystem through the combination of biological and chemical cascade synthesis based on enzyme-modified CPNs and bacteria will provide a new way for augmenting synthesis of valuable chemicals by taking advantage of solar energy.

Compared with protein hydrolysis and chemosynthesis, the microbial synthesis of threonine had become the dominating method because of simple process and low cost [30, 31]. It is well known that the threonine is a precursor of many important chemicals such as 2-oxobutyrate, which its synthetic methods included chemosynthesis and microbial synthesis. However, chemosynthesis is expensive and have many by-products [32], and microbial synthesis still need complex genetic modification to improve the production efficiency of 2-oxobutyrate. In this work, we developed a through the self-assembling of threonine deaminase-modified donor-acceptor conjugated polymer nanoparticles (D-A CPNs) and genetically engineered *Escherichia coli* (*E. coli*). The biohybrid system of *E. coli*/D-A CPNs@Enzyme could both enhance the threonine yield and further transform threonine to 2-oxobutyrate by taking advantage of renewable glucose and solar energy. The photogenerated electrons produced by D-A CPNs@Enzyme were transferred to *E. coli* by redox proteins of flavoprotein (Fp) and cytochrome b (Cyt b) on the bacterial membrane, so the procedure increased the production of intracellular NADPH and threonine. The accumulated threonine was further catalyzed by D-A CPNs@Enzyme to produce 2-oxobutyrate. Relying on the photocatalytic, biological and chemical catalysis, the proposed biohybrid system based on D-A CPNs@Enzyme/bacteria not only realized the synthesis of high value-added chemicals but also provided a brave frontier for the biological application of CPNs and microbial synthesis.

## 2. Results

### 2.1. Design of Organic Semiconductor-*E. coli* Photosynthetic Biohybrid System

**Figure 1:**
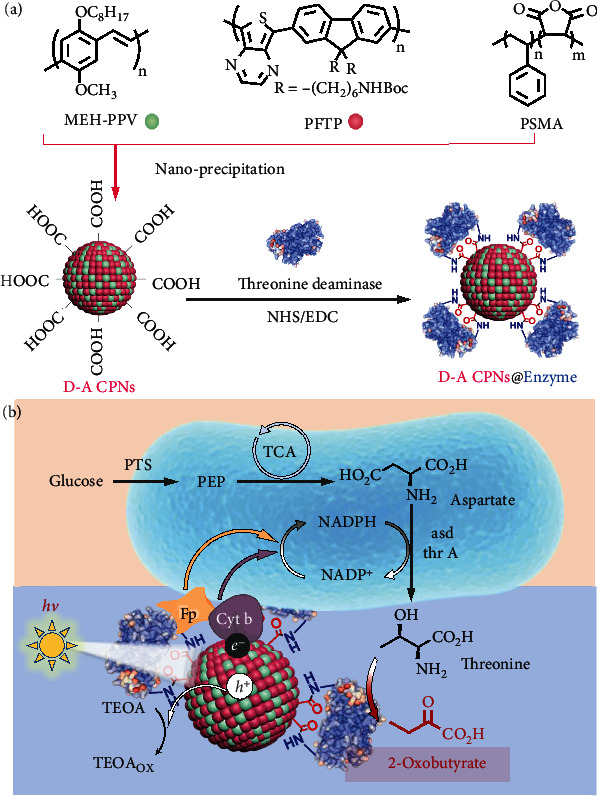
Schematic diagram of the *E. coli*/D-A CPNs@Enzyme biohybrid system. (a) Chemical structures of MEH-PPV, PFTP, and PSMA used for the preparation of D-A CPNs@Enzyme through nanoprecipitation and covalent interactions. (b) The D-A CPNs@Enzyme was incubated with *E. coli* to form *E. coli*/D-A CPNs@Enzyme biohybrid system, also the schematic of the hypothetical electron transfer pathway in which intracellular NADPH regeneration by photoelectrons from D-A CPNs and the synthesis of 2-oxobutyrate. PTS: phosphoenolpyruvate, carbohydrate phosphotransferase system; PEP: phosphoenolpyruvate; TCA: tricarboxylic acid cycle; asd: aspartate semialdehyde dehydrogenase; thr A: aspartate kinase I; thd: threonine deaminase; h^+^: electron hole; e^−^: electron; TEOA: triethanolamine; TEOA_ox_: oxidized triethanolamine.

The *E. coli*/D-A CPNs@Enzyme photosynthetic biohybrid system is constructed by self-assembling of enzyme-modified CPNs and genetically engineered *E. coli*. The engineered *E. coli* producing threonine was selected to construct the biohybrid system. The light-harvesting D-A CPNs were prepared by nanoprecipitation of poly[2-methoxy-5-(2-ethylhexyloxy)-1,4-phenylenevinylene] (MEH-PPV) as electron donor and poly(fluorene-alt-thienopyrazine) (PFTP) as electron acceptor with poly(styrene-co-maleic anhydride) (PSMA), and then, threonine deaminase was linked to D-A CPNs through the amide bond (Figure 1(a)). Under light irradiation, D-A CPNs coating on *E. coli* generate electrons and holes efficiently and the electrons can transfer into *E. coli* to promote the regeneration of intracellular nicotinamide adenine dinucleotide phosphate (NADPH) and further increase the yield of threonine. Moreover, the threonine metabolized from bacteria could be catalyzed into 2-oxobutyrate by threonine deaminase in the presence of cofactor pyridoxal phosphate (Figure 1(b)). Therefore, the proposed biohybrid system *E. coli*/D-A CPNs@Enzyme could capture light irradiation and improve the natural metabolic pathway of *E. coli* to synthesize threonine more efficiently. Then, the augmenting effect of value-added chemical is contributed to the combination of biological and chemical cascade synthesis.

### 2.2. Preparation and Characterization of D-A CPNs@Enzyme Hybrid Nanoparticles

**Figure 2:**
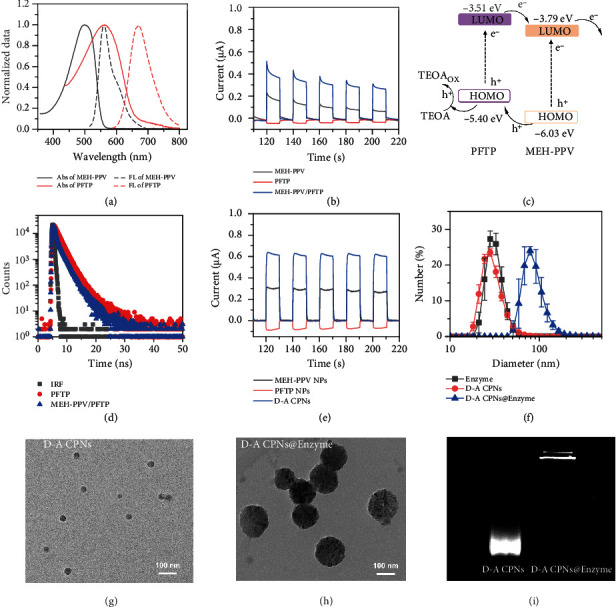
Preparation and characterization of D-A CPNs@Enzyme. (a) Normalized absorption and emission spectra of MEH-PPV and PFTP. (b) Representative photocurrent responses of conjugated polymer. (c) Energy level illustration of MEH-PPV and PFTP. (d) The lifetime decay spectrum of PFTP and MEH-PPV/PFTP by monitoring the emission of 655 nm under an excitation wavelength of 500 nm. IRF was the instrument response function. (e) Representative photocurrent responses of D-A CPNs. (f) Size distribution of D-A CPNs, Enzyme, and D-A CPNs@Enzyme. (g and h) TEM image of D-A CPNs and D-A CPNs@Enzyme, representatively. The scale bar is 100 nm. (i) The agarose gel electrophoresis of D-A CPNs and D-A CPNs@Enzyme.

The carboxylic D-A CPNs were prepared by hydrophobic conjugated polymers (MEH-PPV and PFTP) and amphipathic PSMA with the methods nanoprecipitation as previous work [33, 34]. The threonine deaminase was then linked to D-A CPNs through the condensation reaction of the amino group of threonine deaminase and the carboxyl group on D-A CPNs (Figure 1(a)). As shown in Figure 2(a), MEH-PPV and PFTP exhibited the broad absorption spectra which allowed D-A CPNs to efficiently harvest visible light. To illustrate the ability of charge separation of D-A polymers, photocurrent responses of MEH-PPV, PFTP, and MEH-PPV/PFTP were measured (Figure 2(b)) and the higher photocurrent of MEH-PPV/PFTP was observed. The MEH-PPV and PFTP produced electrons and holes under illumination. As an electron sacrificial donor widely used in photocatalysis, triethanolamine (TEOA) was oxidized by hole to generate TEOA_ox_ [35, 36], which enabled the electron generated by the materials to be transferred to bacteria and inhibited the electron-hole recombination of MEH-PPV and PFTP. Since the LUMO energy level of MEH-PPV was lower than that of PFTP, the photogenerated electrons of PFTP could transfer to the LUMO of MEH-PPV to avoid falling back to the HOMO energy level of PFTP. And the anodic photocurrent was detected by the electrochemical workstation. This could be verified by calculating the energy levels of HOMO and LUMO for two conjugated polymers from ultraviolet photoelectron spectra (UPS) and UV-Vis spectra (Supplementary Figure 1). The difference of HOMO and LUMO energy levels between the two polymers was more than 0.28 eV, which gave enough driving force for the efficient separation of electrons and holes (Figure 2(c)). Furthermore, the fluorescence lifetime of PFTP at 655 nm decreased from 1.83 ns to 1.23 ns after the formation of MEH-PPV/PFTP complex, and those of PFTP NPs and D-A CPNs were 0.46 ns to 0.22 ns, respectively. These results demonstrated that the energy transfer from PFTP to MEH-PPV had occurred. Subsequently, the prepared D-A CPNs exhibited significantly higher anodic photocurrent upon comparing MEH-PPV nanoparticles (MEH-PPV NPs) with PFTP nanoparticles (PFTP NPs) with cathodic photocurrent (Figure 2(e)), indicating that the donor-acceptor structure was beneficial to hole/electron separation. Moreover, the absorption spectrum of D-A CPNs was from 400 nm to 700 nm, which was the overlay effect of the MEH-PPV and PFTP, so the D-A CPNs could perform the visible light capture ability as expected (Supplementary Figure 2b). Also, the photocurrent of D-A CPNs was more stable than those of conjugated polymers themselves. Dynamic light scattering (DLS) was used to determine the size of nanoparticles and D-A CPNs@Enzyme (Figure 2(f) and Supplementary Figure 3). The results showed that the average hydrodynamic diameter of D-A CPNs was 30.4 ± 0.8 nm. The covalent junctions of threonine deaminase changed the size of D-A CPNs, and the hydration particle size of D-A CPNs was increased from 30.4 ± 0.8 nm to 93.2 ± 8.2 nm upon coupling with threonine deaminase. The D-A CPNs and D-A CPNs@Enzyme exhibit approximately uniform spherical shape as shown by transmission electron microscopy (TEM) (Figures 2(g) and 2(h)), and the size coincided with the established DLS results. Meanwhile, zeta potential of D-A CPNs changed from −27.5 ± 1.1 mV to −15.6 ± 0.4 mV after enzyme modification (Supplementary Figure 4). The results of agarose gel electrophoresis could also prove the change. As shown in Figure 2(i), D-A CPNs@Enzyme moved slower than D-A CPNs which indicated that the enzyme was successfully modified on the surface of D-A CPNs.

### 2.3. Preparation and Characterization of *E. coli*/D-A CPNs@Enzyme Biohybrid System

**Figure 3:**
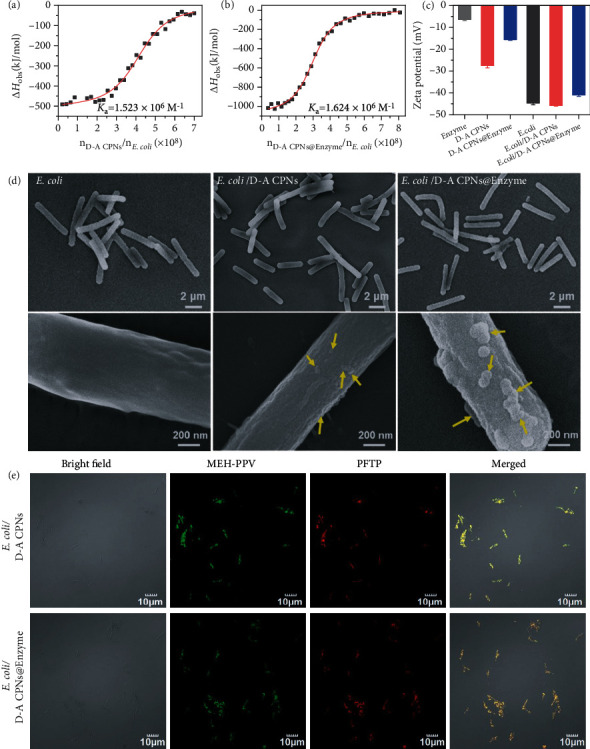
Interaction between D-A CPNs or D-A CPNs@Enzyme and *E. coli*. (a and b) The variation of *ΔH*_obs_ against the injection of *E. coli*/D-A CPNs and *E. coli*/D-A CPNs@Enzyme by titrating D-A CPNs (30 *μ*g/mL) and D-A CPNs@Enzyme (30 *μ*g/mL) into *E. coli* (0.33 OD_600_), respectively. (c) Zeta potentials of enzyme, D-A CPNs, D-A CPNs@Enzyme, *E. coli*, *E. coli*/D-A CPNs and *E. coli*/D-A CPNs@Enzyme, respectively. (d) SEM images of *E. coli*, *E. coli*/D-A CPNs and *E. coli*/D-A CPNs@Enzyme, and the enlarged images of them, respectively. (e) CLSM images of *E. coli* incubated with D-A CPNs, D-A CPNs@Enzyme (10 *μ*g/mL) for 120 min. To avoid the intersection between MEH-PPV and PFTP, the excitation wavelength of MEH-PPV was 488 nm, and the fluorescence image acquisition signal was 490-550 nm. The excitation wavelength of PFTP was 559 nm, and the signal of fluorescence image acquisition was 690-790 nm.

In order to study the interaction mechanism between *E. coli* and D-A CPNs or D-A CPNs@Enzyme, the isothermal titration microcalorimetry (ITC) and zeta potential tests were performed to monitor thermodynamic and surface potential changes. As shown in Figures 3(a) and 3(b), the titration of D-A CPNs or D-A CPNs@Enzyme into *E. coli* suspension obtained S-type enthalpy change (*ΔH*_obs_) curve. The *ΔH*_obs_ changed from negative to zero for *E. coli*/D-A CPNs or *E. coli*/D-A CPNs@Enzyme, which indicated that the interaction of D-A CPNs or D-A CPNs@Enzyme with *E. coli* was an exothermal process. The same interaction was observed between enzyme and bacteria (Supplementary Figure 5). With the continuous addition of D-A CPNs and D-A CPNs@Enzyme, the *ΔH*_obs_ change gradually decreased and finally approached zero, indicating that the binding reached saturation. It is noted that upon adding the D-A CPNs@Enzyme into *E. coli* suspension, the value of *ΔH*_obs_ increased obviously, which indicated that interactions between the enzyme and D-A CPNs with *E. coli* were the synergetic results. Furthermore, the binding constant *K*_a_ was calculated by fitting ITC curves, and the *K*_a_ for *E. coli*/D-A CPNs@Enzyme (1.624 × 10^6^ M^−1^) was higher than those of *E. coli*/D-A CPNs (1.523 × 10^6^ M^−1^) and *E. coli*/Enzyme (6.042 × 10^5^ M^−1^), indicating that D-A CPNs@Enzyme had a stronger affinity towards *E. coli* surface. Meanwhile, the zeta potentials of isolated D-A CPNs, D-A CPNs@Enzyme, and *E. coli* were −27.5 ± 1.1 mV, −15.6 ± 0.3 mV, and −44.7 ± 0.8 mV, respectively (Figure 3(c)). Upon incubating with D-A CPNs or D-A CPNs@Enzyme, the zeta potentials of *E. coli* moved to −45.8 ± 0.4 mV or −41.0 ± 0.6 mV, which was consistent with the results of ITC and proved that binding of D-A CPNs or D-A CPNs@Enzyme towards bacteria. Therefore, we speculated that the dominant driving force of D-A CPNs or D-A CPNs@Enzyme binding to bacteria was the hydrogen bond interactions between carboxyl groups on the D-A CPNs or enzyme surface with negatively charged *E. coli* surface.

The invisible exhibition of the interactions between D-A CPNs or D-A CPNs@Enzyme and *E. coli* was characterized by scanning electron microscopy (SEM) and confocal laser scanning microscopy (CLSM). Compared to the smooth surface of *E. coli*, the surface of *E. coli* incubated with D-A CPNs became significantly rougher and there were particles of approximately 30 nm attached to the surface, indicating that D-A CPNs could evenly coat on the surface of bacteria. Simultaneously, the surface of *E. coli* connected with D-A CPNs@Enzyme had large spherical particles with a diameter of about 100 nm, which suggested that D-A CPNs@Enzyme was coated on the surface of *E. coli* (Figure 3(d)). As shown in Figure 3(e), the CLSM images showed that *E. coli* incubated with D-A CPNs or D-A CPNs@Enzyme exhibited both green and red fluorescence from MEH-PPV and PFTP respectively. It was noted that the green and red fluorescence were well positioned on the surface of bacteria and surrounded the periphery of *E. coli*, indicating that D-A CPNs or D-A CPNs@Enzyme were successfully coated on the cell wall of *E. coli*.

### 2.4. Photosynthesis of *E. coli*/D-A CPNs@Enzyme Biohybrid System

**Figure 4:**
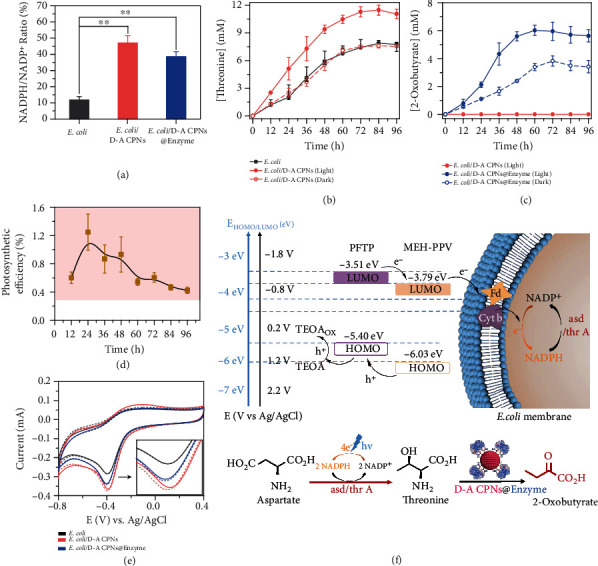
Synthesis, transformation, and electron transfer mechanisms of the *E. coli*/D-A CPNs@Enzyme biohybrid system. (a) The NADPH/NADP^+^ ratio of *E. coli*, *E. coli*/D-A CPNs, and *E. coli*/D-A CPNs@Enzyme under illumination. (b) Threonine concentration of *E. coli* and *E. coli*/D-A CPNs with 40 mM TEOA and 40 mM glucose in the alternating light and dark switch of each 12 hours. (c) 2-Oxobutyrate concentration of *E. coli* and *E. coli*/D-A CPNs@Enzyme with 40 mM TEOA and 40 mM glucose in the alternating light and dark switch of each 12 hours. (d) Estimated external photosynthetic efficiency of threonine production and coupled NADPH regeneration. (e) CVs of the *E. coli*, *E. coli*/D-A CPNs, and *E. coli*/D-A CPNs@Enzyme in anaerobic environment. (The dotted and solid lines were measured under dark and light conditions, respectively.) (f) the energy level diagram of MEH-PPV/PFTP and the electron transport mechanism on the bacterial membrane. ^∗^*P* < 0.05, ^∗∗^*P* < 0.01, and ^∗∗∗^*P* < 0.001 relative to control (Tukey's test). The variation is represented by the standard error of three independent replicates in all data points.

To explore biocompatible conditions for *E. coli*, the toxicity of D-A CPNs and triethanolamine (TEOA) towards *E. coli* at different concentrations as well as the light intensity were optimized. As shown in Supplementary Figure 6, according to the optical density at 600 nm, we finally selected 10 *μ*g mL^−1^ D-A CPNs, 40 mM TEOA, and 2 mW cm^−2^ light intensity for bacteria level experiments. To clarify the photo-enhanced performance of *E. coli*/D-A CPNs@Enzyme biohybrid system, NADPH/NADP^+^ ratio inside *E. coli* was measured. The regeneration of NADPH is the rate-limiting step of microbial production and metabolism [3]. The way to increase the NADPH generation endows the carbon flux in the microbial center to be directed to the desired product [37]. Under light irradiation, increased NADPH/NADP^+^ ratios were obtained from 11.8% to 47.0% and 38.6% for *E. coli*/D-A CPNs and *E. coli*/D-A CPNs@Enzyme, respectively (Figure 4(a)). The NADPH/NADP^+^ ratio of *E. coli/*D-A CPNs@Enzyme was lower than that of *E. coli*/D-A CPNs, which was mainly due to the slight decrease of electron transfer ability of D-A CPNs after the formation of large D-A CPNs@Enzyme particles. Notably, the coating of D-A CPNs or D-A CPNs@Enzyme on *E. coli* surface could promote the regeneration of the cofactor NADPH under light irradiation and also accelerate the conversion of aspartate to threonine and increase the intracellular total protein content (Supplementary Figure 7). The threonine converted from glucose through PEP and TCA by engineered *E. coli* without other carbon sources was identified by high-performance liquid chromatography-mass spectrometry (HPLC-MS) and high-performance liquid chromatography (HPLC) (Supplementary Figure 8 and Supplementary Figure 9). However, due to the lack of NADPH, the process of catalyzing aspartate into threonine through aspartate semialdehyde dehydrogenase and aspartate kinase I became the rate-limiting step for fermentation of threonine. To obtain the maximum threonine yield, we optimized the single carbon source glucose concentration and light intensity (Supplementary Figure 10). High glucose concentration could lead to bacterial growth abundantly and by-product generation from anaerobic respiration, such as acetic acid. The production rate of threonine was the highest under the light intensity of 2 mW cm^−2^. Furthermore, the presence of the threonine deaminase cofactor pyridoxal phosphate in D-A CPNs@Enzyme was verified by mass spectrometry (Supplementary Figure 11a). We screened out the best pH of catalytic performance and also certified that the enzyme activity was not reduced after linking to D-A CPNs (Supplementary Figure 11b, 11c). For simulating the intermittent nature of solar sources, the *E. coli*/D-A CPNs@Enzyme biohybrid system was operated in a light-dark cycle with a light cycle of 12 hours. Under light irradiation with the intensity of 2 mW cm^−2^, the threonine concentration of *E. coli*/D-A CPNs reached the highest amount after growth and fermentation of 72 h (Figure 4(b)). The cumulative threonine concentration of *E. coli*/D-A CPNs (11.3 ± 0.56 mM) under light was 37% higher than that of bare *E. coli* (7.8 ± 0.45 mM). This significant increase was attributed to the effective hole/electron separation for D-A CPNs under light irradiation. The concentration of the produced threonine began to decline after 84 h. It was because that *E. coli* could consume and convert threonine into life-supporting substrates when lacking the required nutrients [38, 39]. As a contrast, the threonine concentration of *E. coli*/D-A CPNs under the dark was only 7.5 ± 0.13 mM. Furthermore, the threonine deaminase in *E. coli*/D-A CPNs@Enzyme system could catalyze the threonine generating from photo-enhanced metabolism of *E. coli* into 2-oxobutyrate (Figure 4(c)).The kinetics of 2-oxobutyrate in *E. coli*/D-A CPNs @Enzyme showed that the conversion of threonine to 2-oxobutyrate reached its maximum value at 72 h. Because the *E. coli* was forced to use 2-oxobutyrate to sustain in the absence of nutrients, the concentration of 2-oxobutyrate was decreased slowly after the point of 72 h [40]. For *E. coli*/D-A CPNs@Enzyme, the produced 2-oxobutyrate concentration was 6.0 ± 0.15 mM under light with an increase of 58% than 3.8 ± 0.18 mM in dark. So, the *E. coli*/D-A CPNs@Enzymebiohybrid system convert rate from glucose to 2-oxobutyrate was around 15% by calculating. Under the same condition, the control group of *E. coli*/D-A CPNs could only produce threonine rather than the 2-oxobutyrate, due to the deserted threonine deaminase. These results confirmed that the *E. coli*/D-A CPNs@Enzyme biohybrid system could not only effectively enhance the production of threonine under light but also convert it into 2-oxobutyrate. In the whole process, the consumption of glucose and the growth of bacteria in the biohybrid system were comparable to those in the control experiments without or D-A CPNs (Supplementary Figure 12), indicating that the increase of threonine production was due to light illumination rather than bacterial proliferation and carbon of source consumption. After 12 h of fermentation, the photosynthetic efficiency of *E. coli*/D-A CPNs reached a maximum of 1.25 ± 0.26% (Figure 4(d)), which was higher than those of plants and algae [41–43].

The electron transport processes of *E. coli*/D-A CPNs and *E. coli*/D-A CPNs@Enzyme were elucidated by cyclic voltammetry (CV) under light and in dark (Figure 4(e) and Supplementary Figure 13). *E. coli* itself exhibited a reduction peak in the range of -0.2 ~ -0.6 V, which may be related to the redox mediator flavoprotein (Fp) and cytochrome b (Cyt b) on the bacterial membrane [44].The reduction current of *E. coli*/D-A CPNs and *E. coli*/D-A CPNs@Enzyme increased significantly, indicating that D-A CPNs improved the electron transfer efficiency. The resistance of *E. coli*/D-A CPNs@Enzyme (38 *Ω*) was higher than *E. coli*/D-A CPNs (26 *Ω*) by electrochemical impedance spectroscopy (EIS) (Supplementary Figure 14). So the reduction currents of *E. coli*/D-A CPNs@Enzyme were lower than *E. coli*/D-A CPNs, which might be caused by the enzyme increasing the resistance and decreasing the conductivity of *E. coli*/D-A CPNs. Furthermore, their reduction currents were reduced under light radiation compared with that of *E. coli*, illustrating the existence of electron transfer between the D-A CPNs and the bacteria. Moreover, the reduction potential of MEH-PPV/PFTP was more negative than Fq and Cyt b on the bacterial surface; thus, Fq and Cyt b could accept electrons from D-A CPNs (Figure 4(f)), which clarified that the photogenerated electrons by D-A CPNs could be effectively transferred to the redox proteins on the membrane. NADP^+^ obtained electrons from Fq and Cyt b to regenerate NADPH, which promoted the production of threonine. The electron transfer between D-A CPNs and bacteria might also be mediated by cell wall extracellular polymeric substances and pili in *E. coli*/D-A CPNs@Enzyme biohybrid system [45].

**Figure 5:**
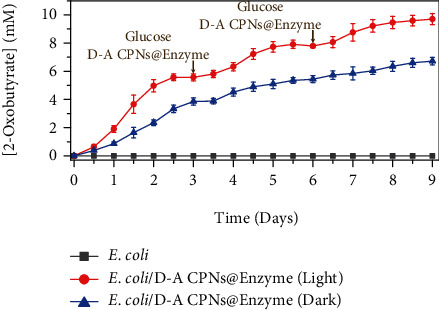
Stability of the *E. coli*/D-A CPNs@Enzyme biohybrid system. The 2-oxobutyrate concentration of *E. coli* and *E. coli*/D-A CPNs@Enzyme with 40 mM TEOA and 40 mM glucose in the alternating light and dark switch of each 12 hours. The concentrations of 20 mM glucose, 10 *μ*g mL^−1^ D-A CPNs@Enzyme, and 20 mM TEOA were added on the 3rd and 6th days.

Under the same conditions, 2-oxobutyrate was synthesized with 40 mM ascorbic acid as the electron sacrifice agent (adjusting pH to 7.0). Finally, the yield of 2-oxobutyrate increased by 23% (Supplementary Figure 15), which proved the universality of photosynthetic biohybrid system. Furthermore, to measure the stability of photosynthetic biohybrid system, glucose and D-A CPNs@Enzyme were supplemented every 3 days, and the yield of 2-oxobutyrate synthesis was monitored for 9 consecutive days (Figure 5). With the replenishment of substrates, the photosynthetic biohybrid system could still produce 2-oxobutyrate after 9 days, which indicated that the photosynthetic system had good operation stability.

## 3. Discussion

In summary, a photosynthetic biohybrid system integrating biological and chemical cascade synthesis was constructed based on self-assembling of enzyme-modified organic semiconductor nanoparticles and engineered bacteria for producing value-added chemicals. The photogenerated electrons entered the cytoplasmic media through the protein on the bacterial membrane, efficiently promoting the 37% increment of threonine production. The produced threonine was simultaneously converted into 2-oxobutyrate by the deaminase covalently linking with D-A CPNs. The 2-oxobutyrate yield under light irradiation is increased by 58% in comparison to that in dark. This work provides new ideas for the utilization of organic semiconductors in biosynthesis systems to improve the production efficiency of both natural products and value-added chemicals by taking advantage of renewable carbon sources and solar energy. The electron transport mechanism and metabolic flux propel the design and implementation of superior biological hybrid systems. With the emergence of genetically engineered microorganisms and a variety of light-harvesting materials, it is possible to produce many valuable products in a modular manner.

## 4. Materials and Methods

### 4.1. Materials and Instruments

The poly(phenylene vinylene) derivative poly[2-methoxy-5-(2-ethylhexyloxy)-1,4-phenylenevinylene] (MEH-PPV), poly(styrene-co-maleic anhydride) (PSMA), N-(3-dimethylaminopropyl)-N'-ethyl-carbodiimide hydrochloride (EDC), N-hydroxysuccinimide (NHS), and the solvent tetrahydrofuran (THF, anhydrous, 99.9%) were all purchased from Sigma-Aldrich (Shanghai, China). Poly(fluorene-alt-thienopyrazine) (PFTP) was synthesized as previous work [46]. All organic solvents were purchased from Beijing Chemical Works and used as received. Toray carbon paper (TGP-H-090), Ag/AgCl (saturated in KCl solution), and platinum wire electrodes were purchased from Shanghai Chuxi Industrial Co., Ltd. The total protein kit and glucose kit were purchased from Sangon Biotech (Shanghai) Co., Ltd. and Rongsheng Biotech (Shanghai) Co., Ltd., representatively. Amplite fluorimetric NADP^+^/NADPH ratio assay kit 15264 was used to measure NADPH/NADP^+^ ratio. All the chemicals used in the experiments were purchased from Innochem, Acros, or Alfa Aesar and used as received. ITC was measured on Model TAM 2277-201 microcalorimetric system (Thermometric AB, Järfälla, Sweden). Electrochemical measurements were carried out with Autolab PGSTAT302N (Metrohm, Switzerland). The optical source was a Xenon fiber optic lamp (CXE-350, Optprco, China). Ultraviolet Photoelectron Spectroscopy (UPS, Axis Ultra Dld) was used to measure the HOMO level. The illumination intensity was adjusted by a radiometer (Photoelectric Instrument Factory of Beijing Normal University). Scanning electron microscopy (SEM) images were measured on a JSM 6700F SEM (Hitachi, Japan). Transmission electron microscopy (TEM) images were recorded on a HT7700 microscope (Hitachi, Japan) operated at 100 kV. The threonine and 2-oxobutyrate were measured by high performance liquid chromatography (HPLC) III 400 MHz HD spectrometer (Waters 2535Q system) and Liquid Chromatograph Mass Spectrometer (LC-MS) Orbitrap Fusion Lumos (Thermo Scientific). UV-Vis absorption spectra were recorded on a JASCO V-550 spectrophotometer. Fluorescence and luminescence emission spectra were collected by a Hitachi F-4500 spectrofluorometer equipped with a xenon lamp excitation source. The dynamic light scattering (DLS) and zeta potential measurements were conducted with Malvern Zetasizer Nano ZS90 (ZEN3600).

### 4.2. Bacterial Strains and Media Composition

*Escherichia coli* (*E. coli*) CICC 20905 was obtained from the China Center of Industrial Culture Collection (CICC). Nutrient Broth (NB) medium containing 10 g/L tryptone, 3 g/L beef extract, and 5 g/L NaCl was used as culture medium. The defined photosynthesis medium (DPM) was 40 mM glucose, 1.8 g/L (NH_4_)_2_SO_4_, 2 g/L KH_2_PO_4_, 1 g/L MgSO_4_•7H_2_O, 0.08 g/L FeSO_4_•7H_2_O, 0.08 g/L MnSO_4_•H_2_O, and 1 g/L sodium citrate. The DPM and triethanolamine (TEOA) solutions were sterilized by passage through a 0.2 *μ*m SFCA filter. The medium and bottles were autoclaved for 20 min at 121°C and cooled to room temperature. Late log cultures were cryopreserved at -80°C with 30% sterilized glycerin as a cryoprotectant.

### 4.3. Preparation of D-A CPNs

MEH-PPV and PFTP doped conjugated polymer nanoparticles (D-A CPNs) were prepared by the nanoprecipitation method as reported previously [33]. In a typical procedure, the MEH-PPV, PFTP, and PSMA were dissolved in dried THF to make stock solutions with concentrations of 0.5 mg/mL, 1 mg/mL, and 2 mg/mL, respectively. 5 mL of THF solution mixture containing MEH-PPV (75 *μ*g/mL), PFTP (75 *μ*g/mL), and PSMA (150 *μ*g/mL) was then quickly dispersed into 15 mL of Milli-Q water under vigorous sonication. Excess THF was removed by rotary evaporation at 37°C. The THF-free D-A CPNs dispersion was filtered through a 0.22 *μ*m cellulose membrane filter, resulting in a 50 *μ*g/mL D-A CPNs solution. As contrast, MEH-PPV nanoparticles (MEH-PPV NPs) and PFTP nanoparticles (PFTP NPs) were prepared with the same method.

### 4.4. Preparation of D-A CPNs@Enzyme

The covalent conjugation of enzyme to D-A CPNs was performed via an amide condensation between carboxyl D-A CPNs and amine-containing threonine deaminase. Briefly, 200 *μ*L of concentrated phosphate buffer (PBS, 0.1 M) was added to 2 mL of carboxylate-presenting D-A CPNs solution (30 *μ*g/mL in Milli-Q water), resulting in a solution in 10 mM PBS. Then, the D-A CPNs solution was treated with 10 *μ*L freshly prepared NHS (5 mg/mL) and 20 *μ*L EDC (5 mg/mL) in PBS, followed by gentle shaking at 30°C for 2 h. 200 *μ*L threonine deaminase (15 mg/mL) was then added to the activated D-A CPNs suspension, and the mixture was shaking at 25°C for 12 h. Uncoupled enzyme and excess NHS and EDC were removed by three washes with PBS buffer (10 mM) using a 100 K Amicon Ultra-4 filter under centrifugation at 3,000 rpm for 3 min.

### 4.5. HOMO and LUMO Measurements

The HOMO level of MEH-PPV and PFTP was obtained by UPS measurements. The optical band gap *E*_g_^OPT^ of polymers is calculated by *E*_g_^OPT^ = hc/*λ* = 1240/*λ*, where *h* is Plank's constant, *c* is the speed of light, and *λ* is the wavelength. The LUMO level of polymers could be calculated by the HOMO and the band gap.

### 4.6. Photocurrent Measurements

After hydrophilic treatment with nitric acid and sodium hydroxide, the carbon paper was cut into 1 cm × 1 cm active sections. The 100 *μ*L hydrophobic polymers (500 *μ*M MEH-PPV, PFTP, and MEH-PPV/PFTP) was dropped to a carbon paper electrode and dried in air. Similarly, 100 *μ*L nanoparticles (50 *μ*g mL^−1^ MEH-PPV NPs, PFTP NPs, and D-A CPNs) was dropped to the carbon paper electrode and dried in air. Electrochemical measurements were measured with a standard three-electrode system. Pt and Ag/AgCl electrodes were used as the counter and reference electrode, respectively. The illumination intensity was 60 mW cm^−2^. The photocurrents were measured at the bias voltage of 0.2 V (vs. Ag/AgCl) under periodic light (10 s) and dark (10 s). The electrolyte was PBS containing 100 mM TEOA (pH = 7.4).

### 4.7. TEM Measurements

The prepared 10 *μ*L D-A CPNs (10 *μ*g mL^−1^) and D-A CPN@Enzyme (10 *μ*g mL^−1^) were dropped on the ultrathin carbon film; then, TEM photography was performed after the drying.

### 4.8. Agarose Gel Electrophoresis

The TAE buffer solution of 0.5% agarose (0.5×) was prepared. After being heated and boiled, the solution was poured into a clean mold and naturally cooled to a gel. The prepared gel was put in the electrophoresis cell and added D-A CPNs and D-A CPNs@Enzyme dispersions with 30% glycerin to the hole, respectively. The gel was applied with the voltage of 120 V for 20 min. After the electrification, imaging was used by gel chromatography.

### 4.9. ITC Measurements

The sample cells were loaded with 600 *μ*L PBS or *E. coli* suspensions (OD_600_ = 0.33), and then, the D-A CPNs (30 *μ*g/mL) and MEH-PPV/PFTP NPs-enzyme solution (30 *μ*g/mL) were injected into the stirred sample cell in amounts of 10 *μ*L via a 500 *μ*L Hamilton syringe controlled by a 612 Thermometric Lund pump until the interaction progress was completed. The stirring rate was 90 rpm with a gold propeller. The binding parameters were obtained by fitting the ITC curves.

### 4.10. Zeta Potential Measurements

The three *E. coli* samples were incubated separately with MEH-PPV NPs (10 *μ*g/mL), PFTP NPs (10 *μ*g/mL), and D-A CPNs solution (50 *μ*g/mL) at 120 min at 30°C. The unbound polymer was removed by centrifuging at 7200 rpm for 3 min. The obtained pellets were washed with ultrapure water and then resuspended in ultrapure water for zeta potential measurements. As control, untreated *E. coli* were incubated in the same conditions.

### 4.11. CLSM Characterization of *E. coli* Incubated with D-A CPNs

Overnight cultured *E. coli* were harvested by centrifuging at 7200 rpm for 3 min and washed with PBS for three times. The supernatant was discarded and the remaining bacteria were resuspended in PBS. Then, the bacteria were incubated separately with D-A CPNs (10 *μ*g/mL) and D-A CPNs@Enzyme (10 *μ*g/mL) at 120 min at 30°C. Unbounded polymer was removed by centrifugation at 7200 rpm for 3 min. The mixtures were washed with deionized water twice and then mounted on a glass slide with a cover slip on top and examined by confocal laser scanning microscopy using 488 nm and 559 nm lasers (FV5&LAMAR).

### 4.12. SEM Measurements

The *E. coli* sample was dropped on clean silicon slices and allowed to evaporate at room temperature. After the specimens were dried, 2.5% glutaraldehyde was added for fixation overnight. Next, 2.5% glutaraldehyde was removed and the specimens were washed with sterile water for 2 times. Ethanol was added in a graded series (5, 10, 20, 30, 50, 70, 90, and 100% for 6 min, respectively) followed by natural drying in the air. After coated with platinum, the specimens were measured by SEM.

### 4.13. Photosynthesis Measurements

To prepare *E. coli*/D-A CPNs and *E. coli*/D-A CPNs@Enzyme hybrids, bacterial species with NB medium were incubated at 30°C, 180 rpm about 12 h. Then, the culture was reinoculated at 30°C for 12 h, *E. coli* were harvested by centrifuging at 7200 rpm for 3 min and washed with PBS for two times. *E. coli* were adjusted OD_600_ to 0.2 for 10 mL DPM for promoting the ability of fermentation about 12 h. Then, the bacteria were adjusted OD_600_ to 0.2 and incubated with the same concentration of *E. coli/*D-A CPNs and *E. coli*/D-A CPNs@Enzyme with DPM at 120 min for 30°C. All photosynthesis measurements were used with DPM. Before photosynthesis, 40 mM TEOA and 40 mM glucose were added to DPM. Then, each bottle was stirred magnetically at 300 rpm and heated to a controlled temperature of 30°C. A Xenon fiber optic lamp with filters larger than 420 nm was employed: with a measured light intensity of 2 mW cm^−2^. The system simulated sunlight with light-dark cycles in 4 days. The dark-treated samples were used as the control. During fermentation, pH was adjusted to 7.0 by adding TEOA. The quantitation of photochemical productions was measured by HPLC.

### 4.14. Measurement of Intracellular Total Protein and NADPH/NADP^+^ Ratio

The *E. coli* were harvested by centrifuging at 7200 rpm for 3 min and washed with PBS for three times and were adjusted OD_600_ to 0.2. The supernatant was discarded and the remaining bacteria were resuspended in PBS. Then, the bacteria were incubated separately with D-A CPNs (10 *μ*g/mL) and D-A CPNs@Enzyme (10 *μ*g/mL) for 120 min at 30°C. After resuspending to the DPM medium, the prepared samples were added 40 mM TEOA and measured under the light intensity of 2 mW cm^−2^ for 30 min. The *E. coli* (5 mL), *E. coli*/D-A CPNs (5 mL), and *E. coli*/D-A CPNs@Enzyme (5 mL) were collected by centrifuging at 7300 rpm for 3 min. The 0.5 mL of cells lysis buffer was added and ultrasound was performed for 30 min. After lysis, the supernatant was extracted by ultrasound. The protein content was measured with the total protein kit. The NADPH/NADP^+^ ratio was measured by an Amplitefluorimetric NADPH/NADP^+^ ratio assay kit.

### 4.15. Quantification of Metabolites

The samples were taken from the fermentation vials and were filtered through a 0.22 *μ*m cellulose membrane filter. Then, HPLC quantified the production of threonine and 2-oxobutyrate. The metabolites were analyzed by HPLC with a UV detector equipped with an XBrigde BEH C18 analytical column (5 *μ*m, 4.6 mm × 250 mm). The system was operated in isocratic mode using 98% acetonitrile: 2% 20 mM NaH_2_PO_4_ (pH = 1.9) as mobile phase at a flow rate of 1 mL min^−1^. Standard curves for each metabolite were constructed with pure standards. For threonine and 2-oxobutyrate, the retention time was observed at around 2.7 minutes and 6 min with a detection wavelength at 210 nm, respectively.

### 4.16. External Photosynthetic Efficiency (PE) Calculations

The optical source was a Xenon fiber optic lamp (CXE-350, Optprco, China), and the measured power density was 2 mW cm^−2^. Taking these together, the external photosynthetic efficiency may be calculated as

(1) $$\begin{array}{c} {\text{NADP}}^{+}+2e^{-}+H^{+}\overset{E.\;coli/\text{D}-\text{A CPNs}@\text{Enzyme}}{⟶}\text{ NADPH}\left(\text{NADPH regeneration}\right),\\ \text{Aspartate}+2\text{NADPH}⟶\text{Threonine}+2{\text{NADP}}^{+}\left(\text{Threonine biosynthesis}\right). \end{array}$$

The factor of 2 accounts for 2e^−^ molecule of aspartate to convert to threonine:

(2) $$\begin{array}{c} \text{Aspartate}+4{\text{e}}^{-}+2{\text{H}}^{+}\overset{E.\;coli/\text{D}-\text{A CPNs}@\text{Enzyme }}{⟶}\text{Threonine}. \end{array}$$

The quantum yield was determined by comparison of the rate of threonine production with the measured photon flux. This is established based on the coupled reaction of NADPH regeneration and threonine production from aspartate, giving the following photosynthetic efficiency (PE) equation:

(3) $$\begin{array}{c} \text{PE}\%=\frac{number\;of\;e^{-}required\;to\;convert\;Aspartate\;to\;Threonine}{total\;incident\;photons}\times 100\%\\ \text{PE}\%=\frac{4\times \text{V}\times \left({\left[C_{t}\right]}_{light}-{\left[C_{t}\right]}_{dark}\right)}{\phi_{\text{ph}}\times \text{t}\times \text{A}}\times 100\%, \end{array}$$

where *C* is the threonine concentration, *V* is the suspension volume, *ϕ_ph_* is equal to 69.2 *μ*mol cm^−2^ hr^−1^, *A* is the area of illumination, and *t* is the reaction time (h).

### 4.17. Stability of the *E. coli*/D-A CPNs@Enzyme Biohybrid System

The *E. coli* in NB medium was cultured at 30°C, 180 rpm about 12 h. Subsequently, to prepare *E. coli*/D-A CPNs@Enzyme hybrids, *E. coli* were adjusted OD_600_ to 0.2 for 10 mL DPM for promoting the ability of fermentation about 12 h. The bacteria were adjusted OD_600_ to 0.2 and incubated with D-A CPNs@Enzyme with DPM at 120 min for 30°C. *E. coli*, which did not interact with the material, was used as the control group. The samples were then added to 40 mM TEOA and 40 mM glucose DPM. Other conditions were the same as those of photosynthesis measurements. Then, 10 *μ*g/mL D-A CPNs@Enzyme and the same volume sterilized water were added to the corresponding bottle on the 3rd and 6th days, respectively. 20 mM glucose and 20 mM TEOA were added on the 3rd and 6th days. The system simulated sunlight with light-dark cycles in 9 days. Dark-treated *E. coli*/D-A CPNs@Enzyme were used as the control. The amount of 2-oxobutyrate synthesized was detected.

## Supplementary Materials

Supplementary Materials Figure S1: size distribution of MEH-PPV NPs (a) and PFTP NPs (b). TEM image of MEH-PPV NPs (a) and PFTP NPs (b). The scale bar is 100 nm. Figure S2: ultraviolet photoelectron spectrometer (UPS) measurement of MEH-PPV (a) and PFTP (b). 10 *μ*L of 1 mg/mL polymer was spun on conductive glass to form a polymer film using a spin coating machine. (c) The UV-visible absorption spectroscopy of MEH-PPV and PFTP. (d) The optical properties, energy levels, and band gaps of the two polymers. Figure S3: zeta potentials of enzyme, D-A CPNs, and D-A CPNs@Enzyme. Figure S4: the variation of *ΔH*_obs_ against the injection of *E. coli*/Enzyme by titrating enzyme into *E. coli*. Figure S5: the optimal experiment of the concentration of D-A CPNs (a) and TEOA (b) incubated with the *E. coli*. (c) The optimal experiment of the light intensity for *E. coli*. Figure S6: the amount of intracellular total protein of *E. coli* and *E. coli*/D-A CPNs under light and dark conditions. Figure S7: HPLC-MS spectrum of standard samples (a) and *E. coli* synthetic products (b). The primary product detected is threonine and the red square marks the peak of sample debris caused by mass spectrometry. Figure S8: HPLC spectrum of standard samples of threonine, 2-oxobutyrate, and mixture. Figure S9: (a) the optimal experiment of the concentration of glucose in DPM. (b) The threonine yield in hybrid system under the different light intensities. Figure S10: (a) ESI mass spectrometry of pyridoxal phosphate in the D-A CPNs@Enzyme. (b) The amounts of threonine deaminase connecting with D-A CPNs and the activity of the D-A CPNs@Enzyme. (c) The optimal experiment of pH of threonine deaminase catalyzing threonine into 2-oxobutyrate. Figure S11: (a) ESI mass spectrometry of pyridoxal phosphate in the D-A CPNs@Enzyme. (b) The amounts of threonine deaminase connecting with D-A CPNs and the activity of the D-A CPNs@Enzyme. (c) The optimal experiment of pH of threonine deaminase catalyzing threonine into 2-oxobutyrate. Figure S12: (a) OD_600_ for 96 hours of cultivation in an alternating light and dark cycle of every 12 h, and legend annotations. (b) Glucose consumption for 96 hours of cultivation. Figure S13: CVs of the carbon paper, D-A CPNs, D-A CPNs@Enzyme, *E. coli*, *E. coli*/D-A CPNs, and *E. coli*/D-A CPNs@Enzyme in anaerobic environment under light condition. Figure S14: EIS of D-A CPNs and D-A CPNs@Enzyme. Figure S15: 2-oxobutyrate concentration of *E. coli* and *E. coli*/D-A CPNs@Enzyme.
