# Supplementary material for: Solar-Driven Producing of Value-Added Chemicals with Organic Semiconductor-Bacteria Biohybrid System
Source: Research (Wash D C). 2022 Mar 23;2022:9834093. doi: 10.34133/2022/9834093 (PMC8972406; doi:10.34133/2022/9834093)
Supplement: Supplementary Materials — Figure S1: size distribution of MEH-PPV NPs (a) and PFTP NPs (b). TEM image of MEH-PPV NPs (a) and PFTP NPs (b). The scale bar is 100 nm. Figure S2: ultraviolet photoelectron spectrometer (UPS) measurement of MEH-PPV (a) and PFTP (b). 10 μL of 1 mg/mL polymer was spun on conductive glass to form a polymer film using a spin coating machine. (c) The UV-visible absorption spectroscopy of MEH-PPV and PFTP. (d) The optical properties, energy levels, and band gaps of the two polymers. Figure S3: zeta potentials of enzyme, D-A CPNs, and D-A CPNs@Enzyme. Figure S4: the variation of ΔHobs against the injection of E. coli/Enzyme by titrating enzyme into E. coli. Figure S5: the optimal experiment of the concentration of D-A CPNs (a) and TEOA (b) incubated with the E. coli. (c) The optimal experiment of the light intensity for E. coli. Figure S6: the amount of intracellular total protein of E. coli and E. coli/D-A CPNs under light and dark conditions. Figure S7: HPLC-MS spectrum of standard samples (a) and E. coli synthetic products (b). The primary product detected is threonine and the red square marks the peak of sample debris caused by mass spectrometry. Figure S8: HPLC spectrum of standard samples of threonine, 2-oxobutyrate, and mixture. Figure S9: (a) the optimal experiment of the concentration of glucose in DPM. (b) The threonine yield in hybrid system under the different light intensities. Figure S10: (a) ESI mass spectrometry of pyridoxal phosphate in the D-A CPNs@Enzyme. (b) The amounts of threonine deaminase connecting with D-A CPNs and the activity of the D-A CPNs@Enzyme. (c) The optimal experiment of pH of threonine deaminase catalyzing threonine into 2-oxobutyrate. Figure S11: (a) ESI mass spectrometry of pyridoxal phosphate in the D-A CPNs@Enzyme. (b) The amounts of threonine deaminase connecting with D-A CPNs and the activity of the D-A CPNs@Enzyme. (c) The optimal experiment of pH of threonine deaminase catalyzing threonine into 2-oxobutyrate. Figure S12: (a) O [file 9834093.f1.docx]

**Supplementary Materials**

**
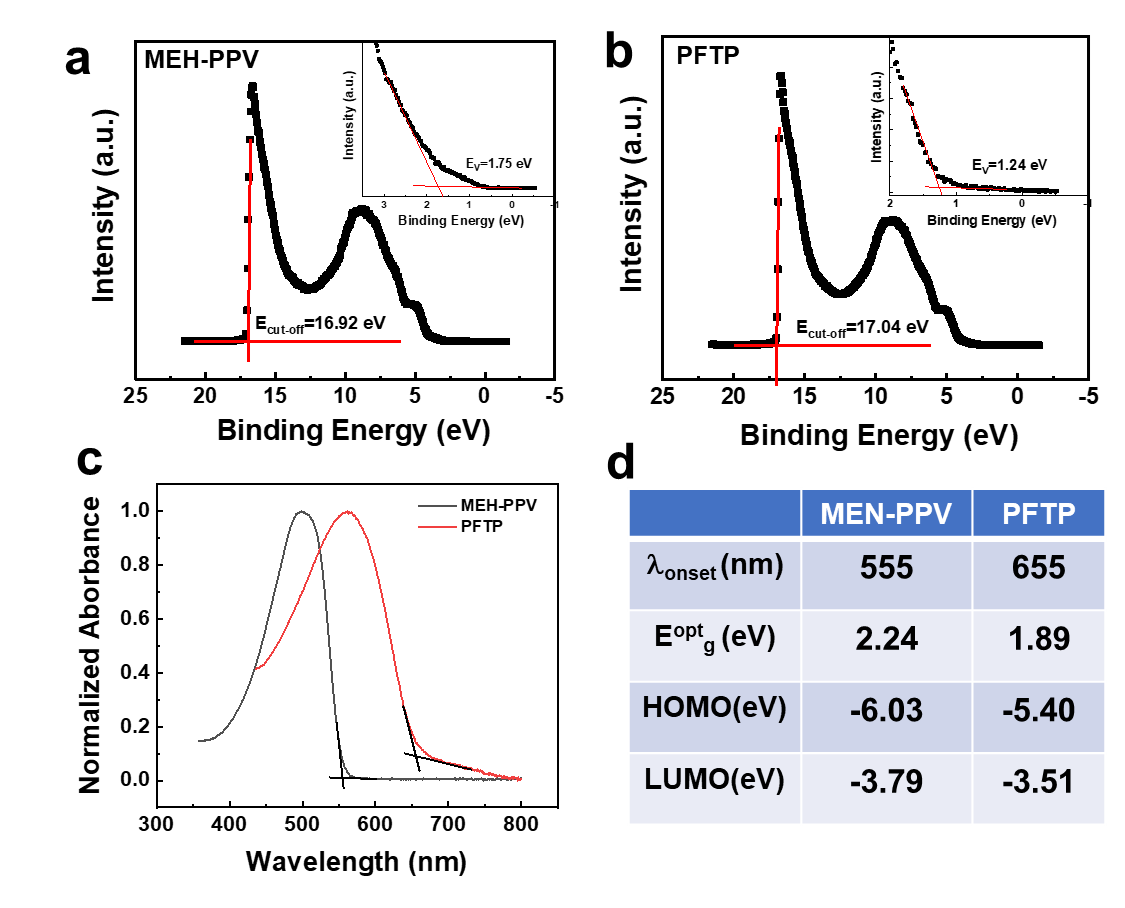
**

**Fig. S1.** Ultroviolet photoelectron spectrometer (UPS) measurement of MEH-PPV (a) and PFTP (b). 10 μL of 1 mg/mL polymer were spun on conductive glass to form a polymer film using a spin coating machine. (c) The UV-visible absorption spectroscopy of MEH-PPV and PFTP. (d) The optical properties, Energy levels and band gaps of the two polymers.

**
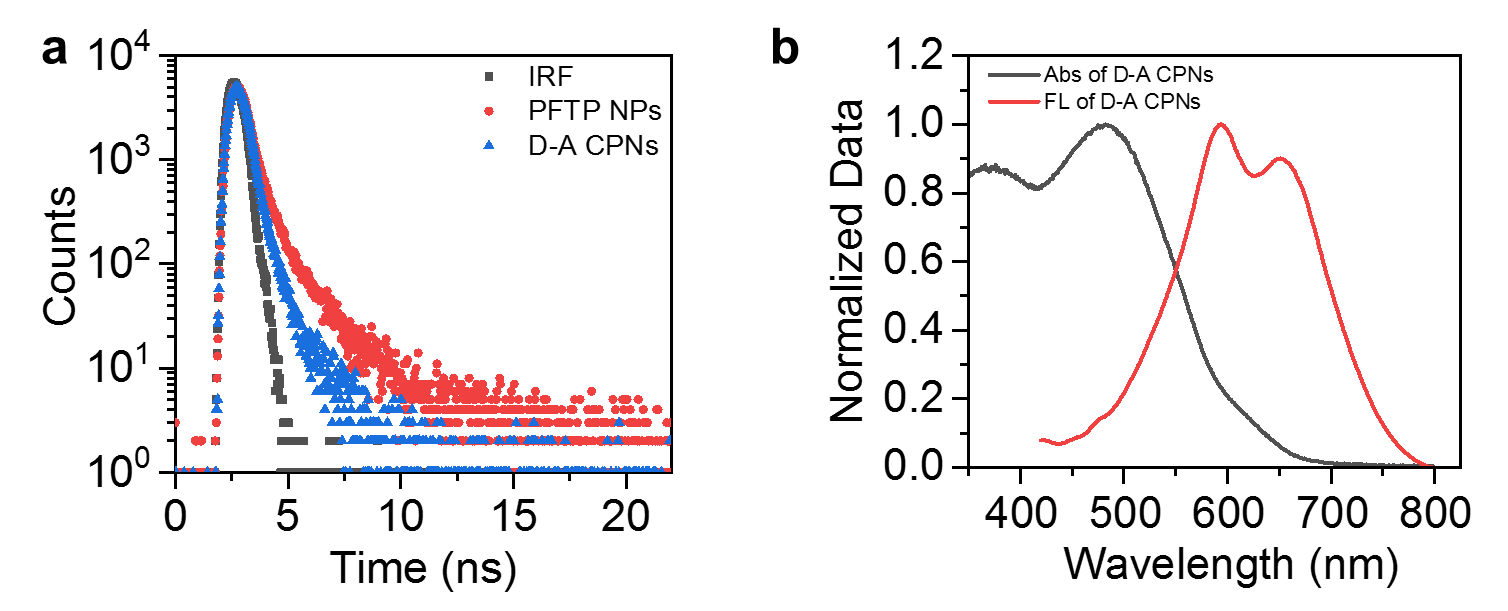
Fig. S2.** (a) The lifetime decay spectrum of PFTP NPs and D-A CPNs by monitoring the emission of 655 nm under an excitation wavelength of 500 nm. IRF was the instrument response function. (b) Normalized absorption and emission spectra of D-A CPNs.


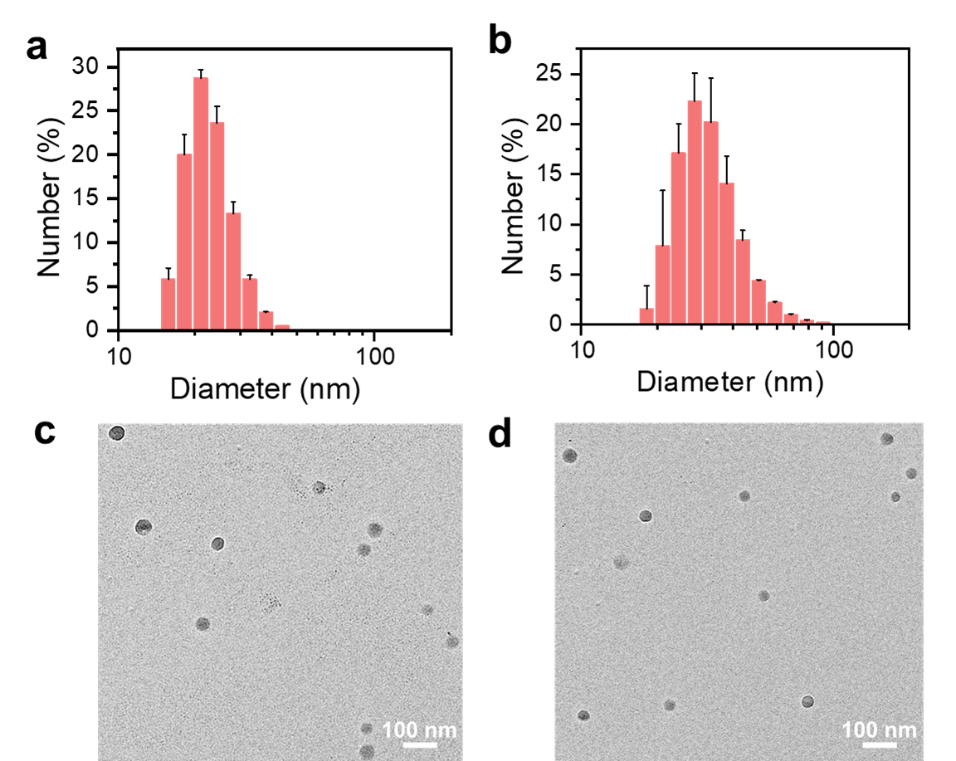


**Fig. S3.** Size distribution of MEH-PPV NPs (a) and PFTP NPs (b). TEM image of MEH-PPV NPs (a) and PFTP NPs (b). The scale bar is 100 nm.

**Fig. S4.** Zeta potentials of enzyme, D-A CPNs and D-A CPNs@Enzyme.


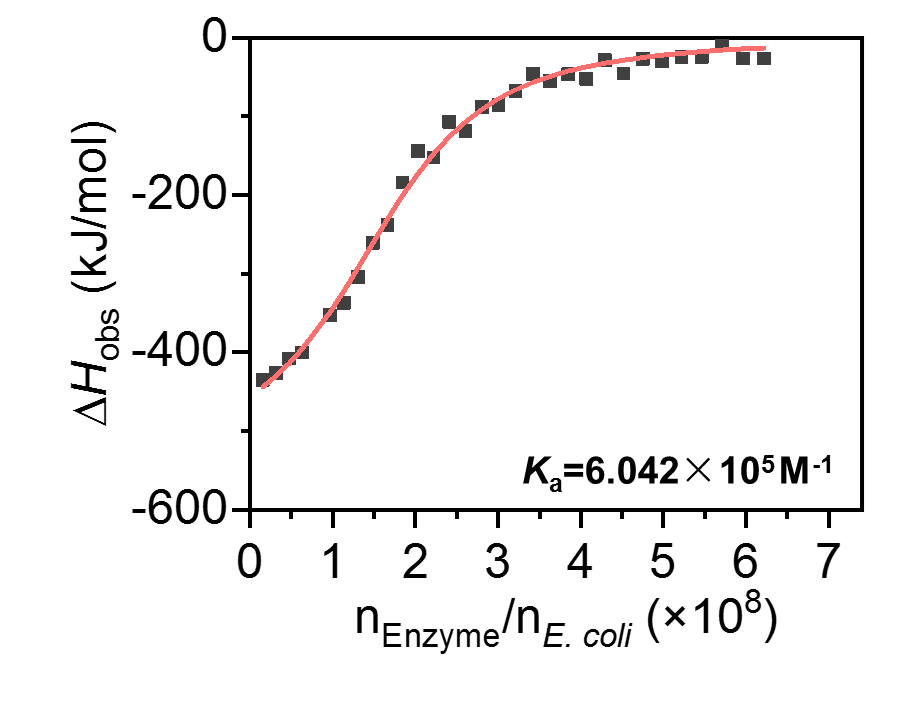


**Fig. S5.** The variation of Δ*H*_obs_ against the injection of *E. coli*/Enzyme by titrating enzyme into *E. coli*.


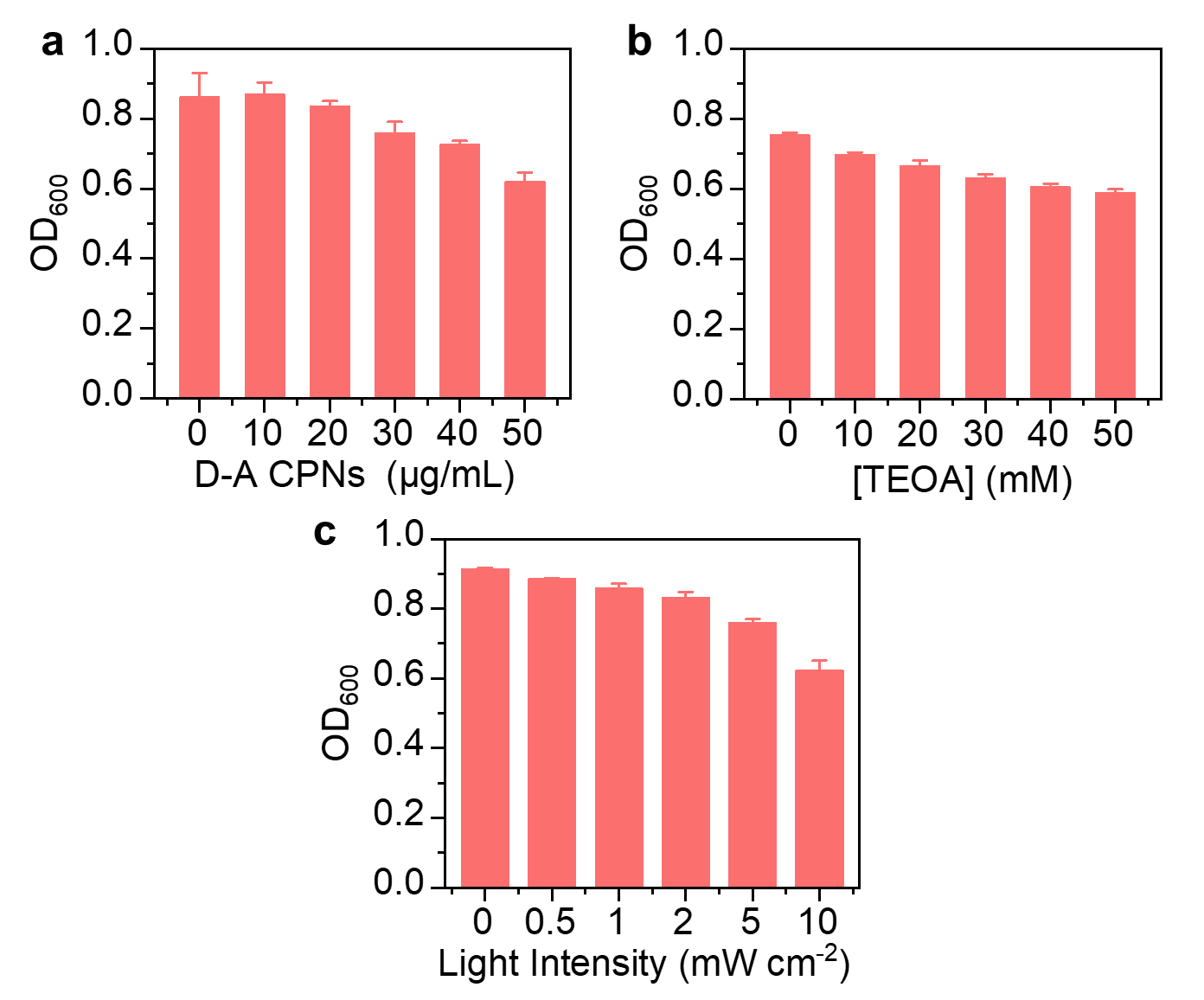


**Fig. S6.** The optimal experiment of the concentration of D-A CPNs (a) and TEOA (b) incubated with *E. coli*. (c) The optimal experiment of the light intensity for *E. coli*.

**
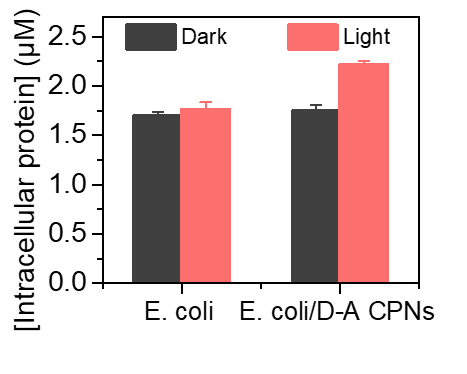
**

**Fig. S7.** The amount of intracellular total protein of *E. coli* and *E. coli*/D-A CPNs under light and dark conditions.


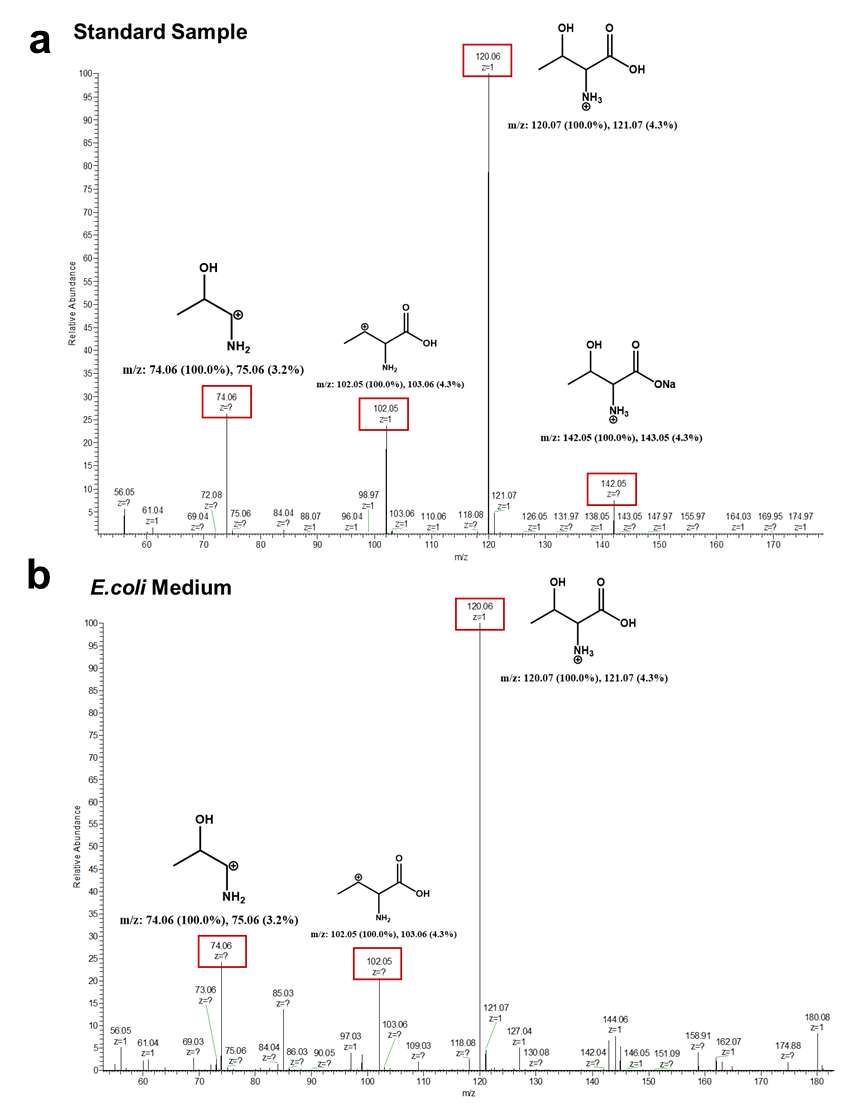


**Fig. S8.** HPLC-MS spectrum of standard samples (a) and *E. coli* synthetic products (b). The primary product detected is threonine and the red square marks the peak of sample debris caused by mass spectrometry.

**Fig. S9.** HPLC spectrum of standard samples of threonine, 2-oxobutyrate and mixture.

**
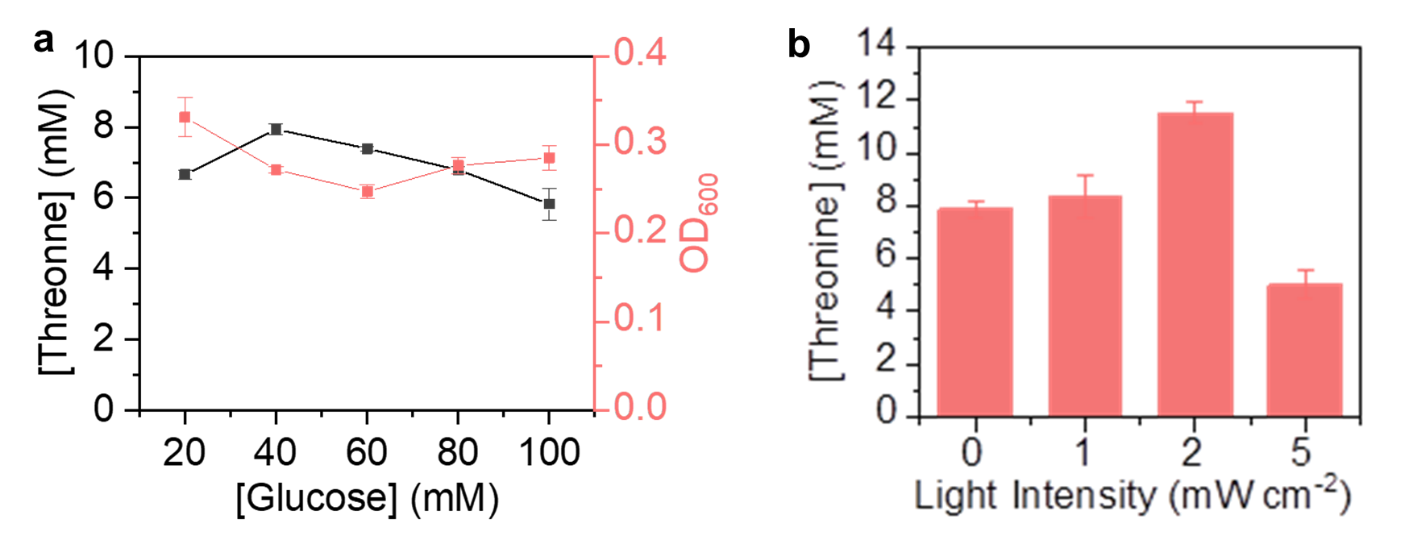
**

**Fig. S10.** (a) The optimal experiment of the concentration of glucose in DPM. (b) The threonine yield in hybrid system under the different light intensities.


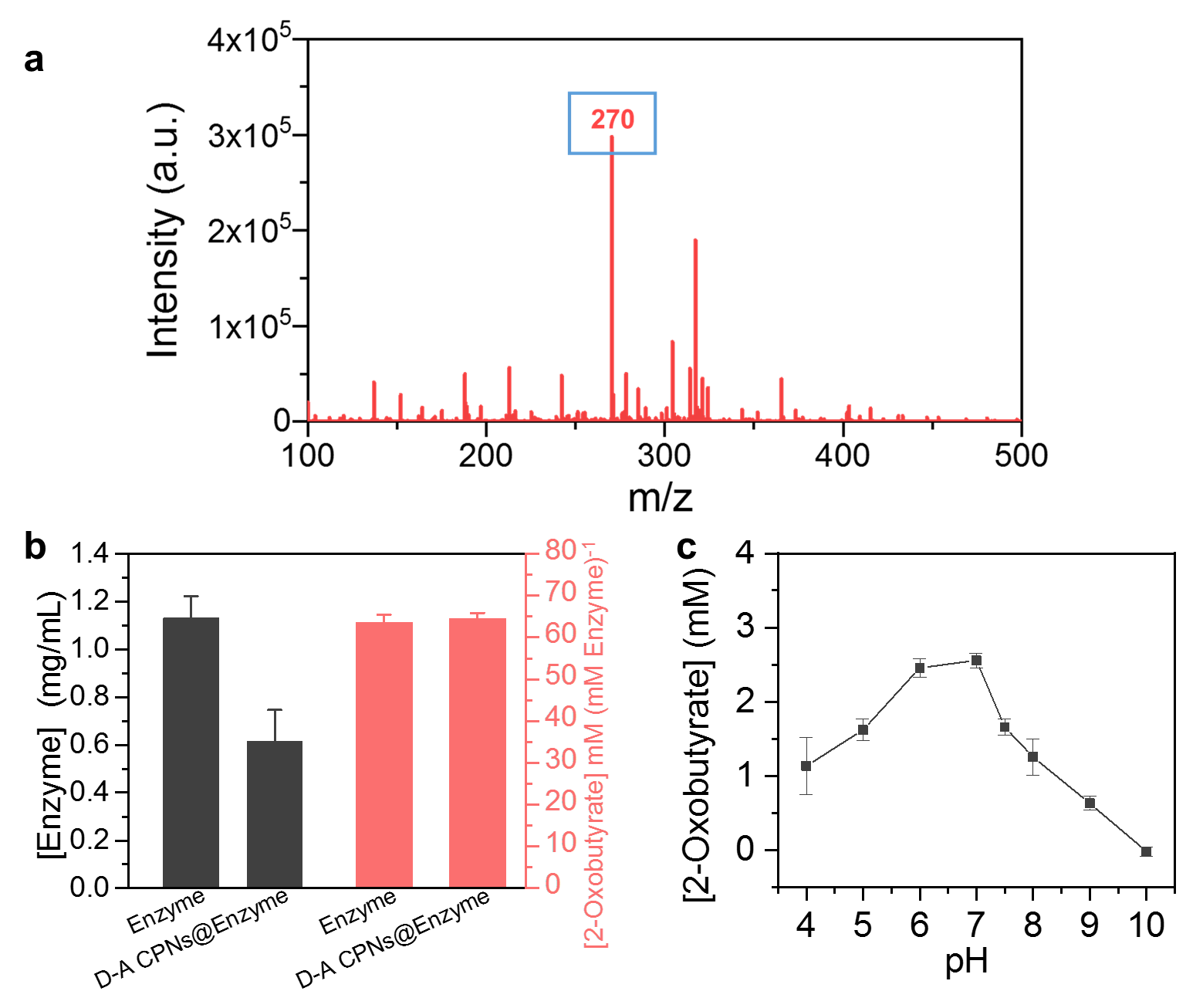


**Fig. S11.** (a) ESI mass spectrometry of pyridoxal phosphate in the D-A CPNs@Enzyme. (b) The amounts of threonine deaminase connecting with D-A CPNs and the activity of the D-A CPNs@Enzyme. (c) The optimal experiment of pH of threonine deaminase catalyzing threonine into 2-oxobutyrate.

**
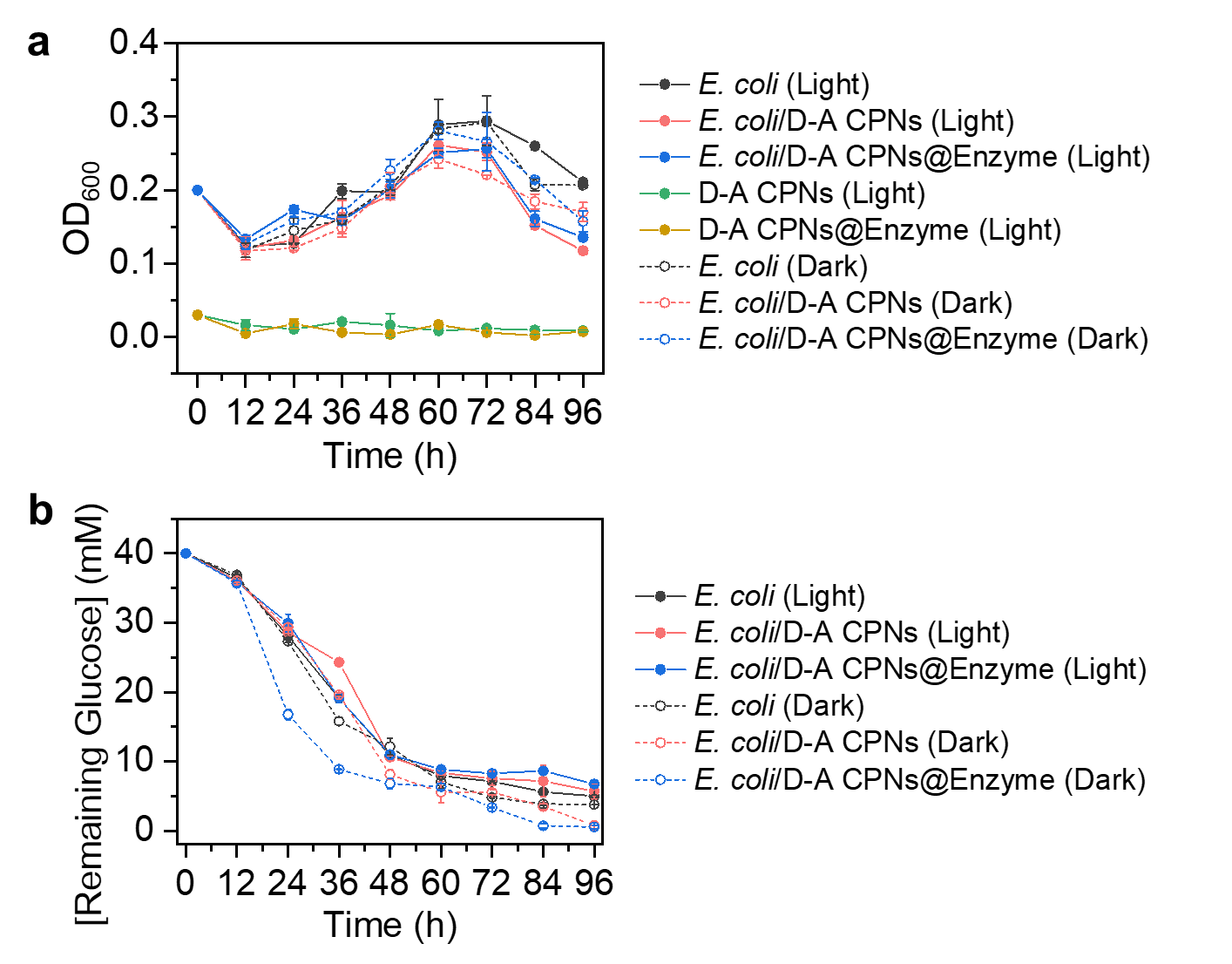
**

**Fig. S12.** (a) OD_600_ for 96 hours of cultivation in an alternating light and dark cycle of every 12 h, and legend annotations. (b) Glucose consumption for 96 hours of cultivation.

**
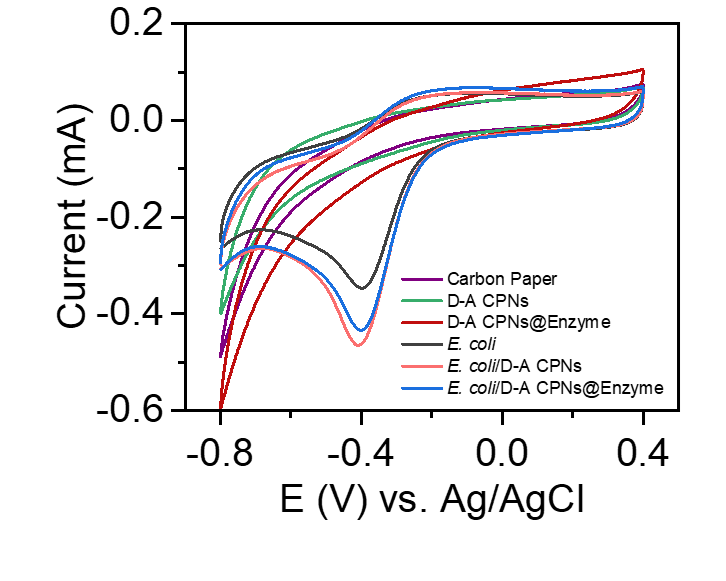
**

**Fig. S13.** CVs of the carbon paper, D-A CPNs, D-A CPNs@Enzyme, *E. coli*, *E. coli*/D-A CPNs and *E. coli*/D-A CPNs@Enzyme in anaerobic environment under light condition.

**Fig. S14.** EIS of D-A CPNs and D-A CPNs@Enzyme. EIS was measured with an Autolab PGATAT302N electrochemical workstation (the electrolyte was 1 mM Fe(CN)_6_^4-^/^3-^ solution). 30 μg/mL D-A CPNs and 30 μg/mL D-A CPNs@Enzyme coated on carbon papers (1 cm^2^) were used as working electrodes. Pt and Ag/AgCl electrode were used as the counter and reference electrode, respectively.


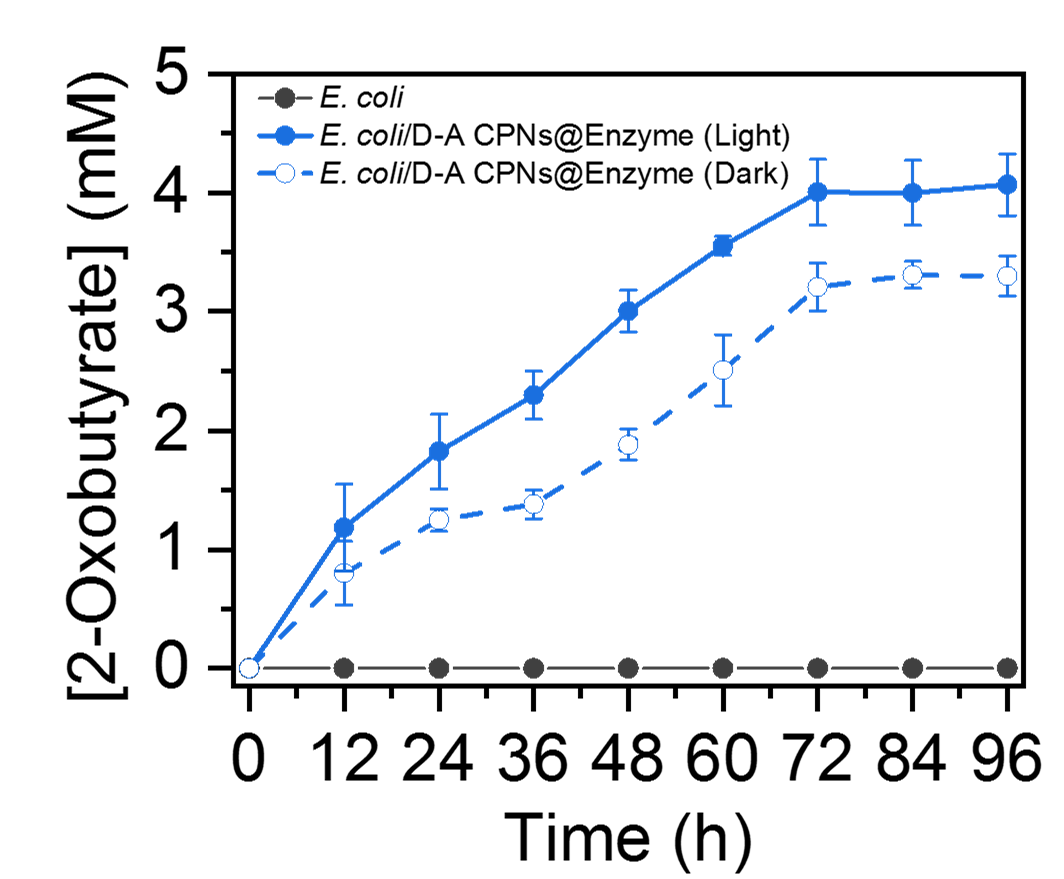


**Fig. S15.** 2-Oxobutyrate concentration of *E. coli* and *E. coli*/D-A CPNs@Enzyme.
